# Genetic Structure and Selective Signature Analysis of Xinjiang Local Sheep Populations

**DOI:** 10.3390/ani16060985

**Published:** 2026-03-21

**Authors:** Chunyan Luo, Marzia Yasen, Feng Bai, Geng Hao, Aminiguli Abulaizi, Lijuan Yu, Nazakaiti Ainivaner, Xinmin Ji, Yuntao Zhang, Jianguo Yu, Yanhua Zhang

**Affiliations:** 1 Key Laboratory of Livestock and Poultry Resources (Sheep) Evaluation and Utilization, Ministry of Agriculture and Rural Affairs, Xinjiang Uygur Autonomous Region Academy of Animal Sciences, Urumqi 830011, China; 18284009137@139.com (C.L.);; 2 College of Animal Science, Xinjiang Agricultural University, Urumqi 830052, China

**Keywords:** whole-genome resequencing, Xinjiang indigenous sheep, sheep, population genetic structure, selective signature analysis, environmental adaptation, economic traits

## Abstract

The unique ecological gradients of Xinjiang have fostered a rich reservoir of genetic resources in local sheep populations. However, the population genetic structure, adaptive mechanisms to extreme environments, and the genetic basis underlying key economic traits of these breeds remain poorly understood. To address this gap, we performed whole-genome resequencing of 140 individuals from seven indigenous sheep populations—Altay, Bayinbuluke, Kazakh, Kirgiz, Bashibai, Turpan Black, and Yemule White—identifying 18,700,507 high-quality SNPs. Genetic diversity analyses revealed that all populations exhibited comparable levels of genetic diversity, with modest variation across breeds, with Turpan Black sheep exhibiting the highest observed heterozygosity (Ho = 0.3110) and proportion of polymorphic sites, whereas Kirgiz sheep showed comparatively lower values. Population structure analyses consistently indicated that geographic isolation is the primary driver of genetic differentiation, with Kirgiz sheep from the Pamir Plateau in southern Xinjiang displaying the greatest genetic distance relative to northern Xinjiang populations. By integrating multiple selection signature detection methods—including F_ST, π ratio, and XP-CLR—we found that genes under selection in Kirgiz sheep were significantly enriched in biological pathways related to stem cell pluripotency regulation (e.g., BMPR1B), DNA repair (e.g., DDB2), and neural development, thereby elucidating their unique genetic adaptations to high-altitude environments. In contrast, Turpan Black sheep appear to cope with heat stress through mechanisms involving basal transcriptional regulation (e.g., GTF2I), maintenance of protein homeostasis (e.g., DNAJB14), and melanin biosynthesis (e.g., MC1R). Furthermore, comparative analysis of body size identified a suite of candidate genes associated with growth and development (e.g., CUX1, KIT), which are primarily involved in transcriptional regulation, protein kinase activity, and the ubiquitin-mediated proteolytic system, thereby revealing a multi-layered genetic regulatory network governing body conformation. Collectively, this study provides a comprehensive genomic framework for understanding the genetic structure, adaptive evolution, and molecular basis of economically important traits in indigenous sheep breeds from Xinjiang, offering valuable candidate targets for future functional validation and precision breeding programs.

## Simple Summary

Xinjiang harbors numerous local sheep breeds that have adapted to extreme environments, including high-altitude plateaus and hot deserts; however, their genetic background remains poorly understood. In this study, we analyzed whole-genome sequences from 140 individuals representing seven indigenous Xinjiang breeds to investigate their genetic diversity, population structure, and the genetic basis of their adaptations to harsh environments and variation in body size. All breeds exhibited comparable levels of genetic diversity, and geographic isolation likely contributed significantly to the observed genetic differentiation among populations. Kirgiz sheep from the Pamir Plateau harbor unique genetic variants in genes involved in DNA repair, energy metabolism, and nervous system development—traits that may facilitate survival under high-altitude conditions. Turpan Black sheep, which are adapted to extreme heat, carry genetic variants potentially associated with heat stress response and coat pigmentation. We also identified several candidate genes linked to body size that may influence growth and development. These findings provide valuable genomic insights to support future breeding programs and conservation efforts for Xinjiang’s indigenous sheep breeds.

## 1. Introduction

As one of the earliest livestock species domesticated by humans, sheep not only constitute a cornerstone of the animal husbandry economy but also serve as an ideal model for investigating adaptive evolution and the genetic architecture of complex traits [1]. Diverse ecological environments across the globe have shaped extensive phenotypic variation in sheep [2]. Owing to its distinctive ecological gradients and rich pastoral traditions, Xinjiang, China, has emerged as one of the world’s most important reservoirs of sheep genetic diversity, harboring substantial genetic variation and pronounced phenotypic heterogeneity [3]. Currently, Xinjiang is home to 12 indigenous sheep breeds, which exhibit a suite of adaptive traits, including cold tolerance, drought resistance, and adaptability to low-quality forage [4]. However, the fine-scale genetic structure, differentiation patterns, and underlying adaptive genetic mechanisms among these local populations remain poorly understood, thereby hindering the precise conservation and efficient utilization of germplasm resources. Therefore, systematically elucidating the genetic differentiation patterns and molecular basis of environmental adaptation in local sheep populations from Xinjiang is of significant theoretical and practical significance for promoting the sustainable utilization of local germplasm resources [5,6].

In recent years, advances in population genomics have established whole-genome resequencing-based selection signature analysis as a powerful approach for dissecting the genetic basis of environmental adaptation and economically important traits in livestock [7,8,9]. For example, in East Adriatic sheep, NOL8 and SYVN1 contribute to adaptation to Mediterranean environments by modulating energy expenditure and maintaining cellular homeostasis under stressful conditions [10], whereas PCDH9 in Romanov sheep from the upper Volga region has been implicated in cold adaptation [11]. Similarly, significant progress has been made in identifying genes underlying key economic traits: PDGFD and BMP2 are associated with tail fat deposition, whereas HOXB13 is widely recognized for its role in skeletal development and body conformation [12,13]. Collectively, these studies underscore that integrating multiple genome-wide selection signature detection methods represents a robust strategy for unraveling the genetic mechanisms underlying both adaptive evolution and economically relevant traits in sheep.

Nevertheless, comprehensive genome-wide investigations of the diverse sheep breeds inhabiting Xinjiang—a classic ecological transition zone—remain scarce. To date, no study has systematically compared the genetic architectures of sheep breeds from northern and southern Xinjiang while simultaneously deciphering their multidimensional adaptive strategies and the genomic basis of key production traits. To bridge this gap, we analyzed whole-genome resequencing data from seven representative indigenous sheep populations distributed across Xinjiang. By integrating population structure inference with multiple complementary selection signature scans (e.g., F_ST, π ratio, XP-CLR), we characterized genome-wide differentiation patterns and identified candidate genes and biological pathways associated with local environmental adaptation and body size variation. This work provides a foundational genomic resource for the molecular evaluation of Xinjiang’s native sheep germplasm and delivers actionable insights—together with prioritized molecular targets—for breed improvement and conservation efforts.

## 2. Materials and Methods

### 2.1. Experimental Materials

**Figure 1:**
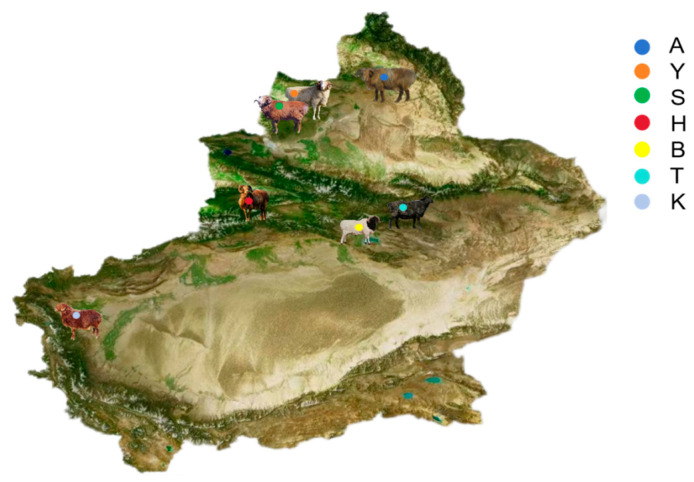
Geographic distribution of the seven sheep populations.

Samples were collected from seven representative indigenous sheep breeds (populations) in Xinjiang. Jugular venous blood (10 mL) was randomly collected from 140 one-year-old individuals with presumed no close kinship based on farmer interviews and random sampling within each flock, with a balanced sex ratio (70 males and 70 females). Blood samples were collected into EDTA-containing anticoagulant tubes, labeled with unique identifiers, immediately placed in insulated containers with ice packs (0–4 °C) in the field, and subsequently transported to the laboratory for long-term storage at −80 °C. Sampling locations spanned a typical ecological gradient across Xinjiang, ranging from northern regions (Altay and Tacheng) to southern Xinjiang (Kizilsu Kirgiz Autonomous Prefecture) (Figure 1), thereby ensuring broad geographic representation. Detailed information on breed names, abbreviations, sampling locations, and sample sizes is provided in Table 1. For brevity, the seven populations are hereafter referred to by the following abbreviations throughout the text: Altay sheep (A), Bayinbuluke sheep (B), Kazakh sheep (H), Kirgiz sheep (K), Bashibai sheep (S), Turpan Black sheep (T), and Yemule White sheep (Y).

### 2.2. DNA Extraction and Whole-Genome Sequencing

Whole-genome DNA was extracted from blood samples using a commercial DNA extraction kit (Beijing Ailede Biotechnology Co., Ltd., Beijing, China). The concentration, integrity, and purity of the extracted genomic DNA were assessed using an Agilent 5400 system (Agilent Technologies, Santa Clara, CA, USA) following the standard operating procedures of the sequencing facility. The quality acceptance criteria required a total DNA amount ≥ 25 ng, with DNA fragments predominantly concentrated above 1000 bp, and no or only slight coloration, viscosity, or extra bands. All 140 samples met these quality criteria. Samples were then subjected to high-throughput paired-end sequencing (PE150) on an Illumina NovaSeq 6000 platform (Illumina, San Diego, CA, USA) at Beijing Novogene Bioinformatics Technology Co., Ltd. (Beijing, China). Each library insert was sequenced from both ends, achieving an average sequencing depth of approximately 10× per individual [14]. Based on the sequencing data, the per-sample depth ranged from 8.29× to 22.49× (SD = 2.8×) across all 140 individuals, and raw sequencing data were generated accordingly.

### 2.3. Sequencing Data Quality Control

Upon obtaining raw short-read sequence (raw reads) data in fastq format, fastp was used to perform quality control on the paired-end sequencing files with the parameters: -z 6 -g -q 5 -u 50 -n 15, removing adapter sequences, low-quality bases, and undetermined bases. The total raw data volume was 4839.58 Gb, with an average of 34.57 Gb per individual. After QC, the total clean data volume was 4766.39 Gb, with an average of 34.05 Gb per individual, representing an average retention rate of approximately 98.5%.

### 2.4. Reference Genome Alignment

After quality control, the BWA (version 0.7.17) software was used to align the high-quality sequencing reads to the sheep reference genome (ARS-UI_Ramb_v3.0; assembly accession GCF_016772045.2). The alignment was performed using the MEM algorithm with parameters -t 4 -k 32 -M. The alignment files were sorted using SAMtools (version 0.1.19), and duplicate reads were removed using the rmdup function in SAMtools.

### 2.5. Variant Detection and Annotation

SAMtools and BCFtools were used for population-level SNP detection (samtools-1.3.1 mpileup -q 1 -C 50 -t SP -t DP -m 2 -F 0.002; bcftools-1.4 call -mv -f GQ). After filtering, high-quality SNPs were retained for subsequent analysis. Finally, ANNOVAR [15] was used to perform functional annotation of the detected genetic variants.

### 2.6. Population Genetic Diversity Analysis

Genetic diversity within populations was primarily assessed using the following metrics: observed heterozygosity (Ho), expected heterozygosity (He), and polymorphic nucleotide proportion (PN). These metrics were calculated using PLINK (version 1.90) with default parameters based on variant site information (--hardy for Ho and He, and --freq for PN).

### 2.7. Population Genetic Structure Analysis

Principal Component Analysis (PCA) was performed using GCTA (version 1.94.1) to compute the genetic covariance matrix based on a subset of SNPs pruned for linkage disequilibrium (LD). The first three principal components were retained and visualized. Phylogenetic relationships were inferred using the neighbor-joining method. Pairwise genetic distances at the individual level were calculated using TreeBest software (version 1.9.2) based on all high-quality SNPs, and the phylogenetic tree was constructed using the neighbor-joining method. The genetic differentiation index (F_ST) was calculated using VCFtools (version 0.1.17) to assess differentiation among populations. Population structure was inferred using ADMIXTURE (v1.3.0), with the number of assumed ancestral population number (K) ranging from 2 to 8, and the optimal K value determined by minimizing cross-validation error. All results were visualized using Python (version 3.12.2) with the Matplotlib (version 3.9.2) and Seaborn (version 0.13.2) libraries for plotting, and Pandas (version 2.2.2) for data processing and manipulation.

### 2.8. Selective Signature Analysis

Three complementary selection signature detection methods—F_ST, π ratio, and XP-CLR—were integrated to perform a genome-wide scan for selective sweeps using 100 kb sliding windows with a 50 kb step size. Specifically, F_ST and π ratio were calculated using VCFtools with the parameters --fst-window-size 100,000 --fst-window-step 50,000 and --window-pi 100,000 --window-pi-step 50,000 respectively. In contrast, XP-CLR was applied using non-overlapping 100 kb windows to enhance mapping resolution [16,17]. To identify genomic regions under strong positive selection, candidate regions were defined as those within the top 5% of the empirical distribution for both F_ST and XP-CLR and within the bottom 5% for π ratio, reflecting reduced nucleotide diversity. Finally, the intersection of candidate regions identified by all three methods was designated as high-confidence consensus selective sweep intervals for downstream functional and evolutionary analyses.

### 2.9. Gene Enrichment

Protein-coding sequences from the sheep reference genome (GCF_016772045.2; file: GCF_016772045.2_ARS-UI_Ramb_v3.0_protein.faa) were annotated using the online version of eggNOG-mapper, generating annotation files containing Gene Ontology (GO) terms and KEGG Orthology (KO) identifiers. Subsequently, GO and KEGG pathway enrichment analyses were performed for genes located within the candidate selective regions using TBtools-II (version 2.376).

## 3. Results

### 3.1. SNP Detection

**Figure 2:**
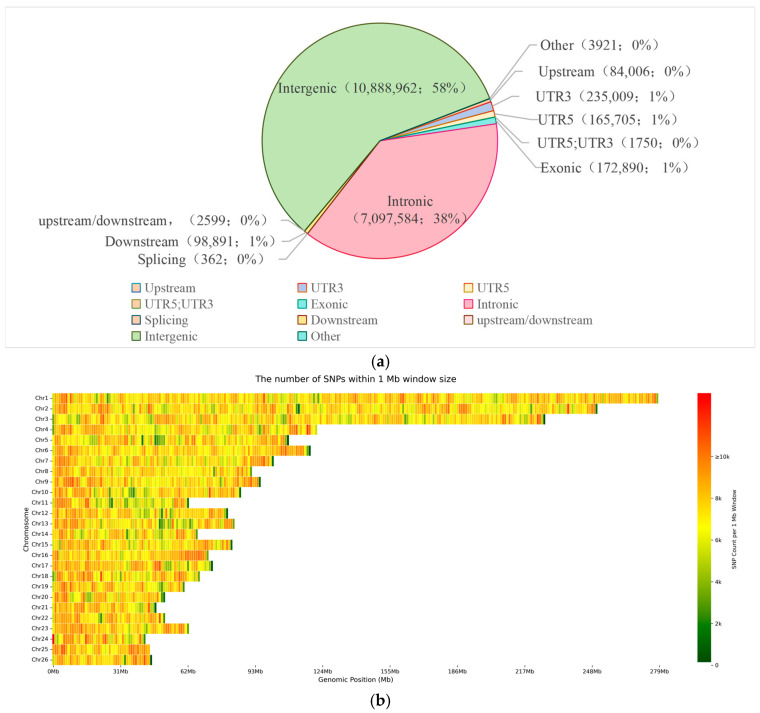
(**a**) Functional annotation of genomic SNPs; (**b**) Distribution of SNPs across chromosomes.

A total of 44,755,989 SNPs were identified across 140 sheep using SAMtools and related software. After filtering, 18,700,507 high-quality SNPs were retained, including 84,006 located in gene upstream regions, 172,890 in exonic regions, and 7,097,584 in intronic regions (Figure 2a). The chromosomal distribution of SNPs was visualized as a genome-wide distribution map (Figure 2b), which showed the highest absolute SNP counts on chromosomes 1 and 2, consistent with their larger physical sizes.

### 3.2. Population Genetic Diversity

**Figure 3:**
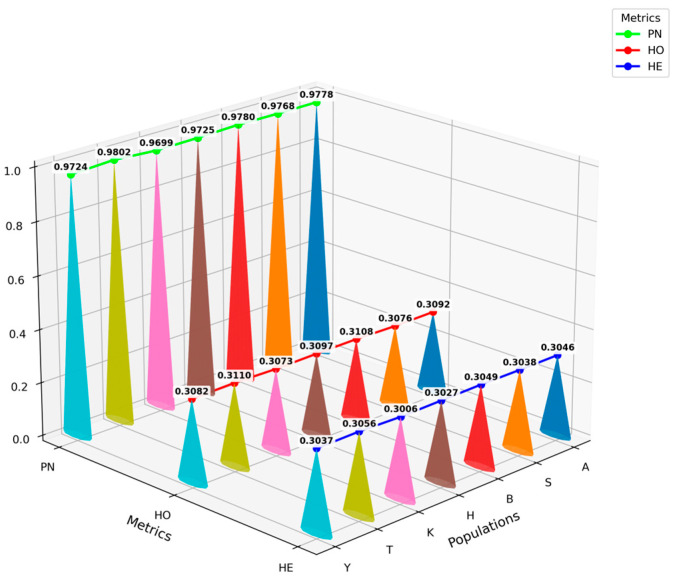
Genetic diversity parameters of the seven populations.

Genetic diversity metrics for the seven sheep populations are summarized in Figure 3. Across all populations, observed heterozygosity (Ho) marginally exceeded expected heterozygosity (He). The detailed diversity estimates for each population were as follows: Altay sheep (Ho = 0.3092 ± 0.1686, He = 0.3046 ± 0.1477), Bayinbuluke sheep (Ho = 0.3108 ± 0.1694, He = 0.3049 ± 0.1475), Kazakh sheep (Ho = 0.3097 ± 0.1720, He = 0.3027 ± 0.1497), Bashibai sheep (Ho = 0.3076 ± 0.1690, He = 0.3038 ± 0.1484), Turpan Black sheep (Ho = 0.3110 ± 0.1683, He = 0.3056 ± 0.1466), Kirgiz sheep (Ho = 0.3073 ± 0.1720, He = 0.3006 ± 0.1512), and Yemule White sheep (Ho = 0.3082 ± 0.1707, He = 0.3037 ± 0.1490). The proportion of polymorphic sites ranged from 0.9699 to 0.9802, with Kirgiz sheep at the lower end and Turpan Black sheep at the upper end of this range. Overall, Turpan Black sheep ranked highest across all three diversity indices, whereas Kirgiz sheep ranked lowest, reflecting nominally lower diversity. Nevertheless, the magnitude of differences among populations was modest, with all values falling within a narrow range, indicating overall similarity in genetic variability.

### 3.3. Principal Component Analysis (PCA)

**Figure 4:**
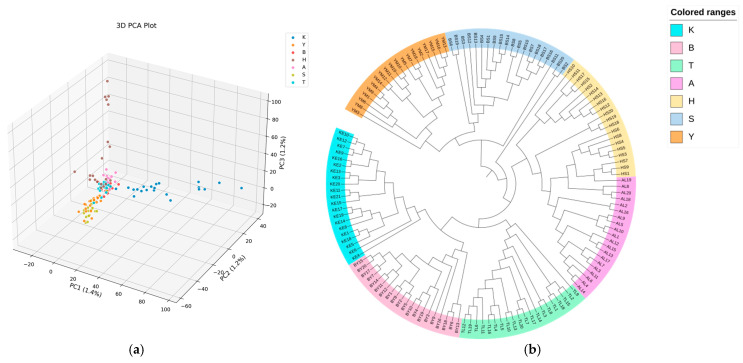

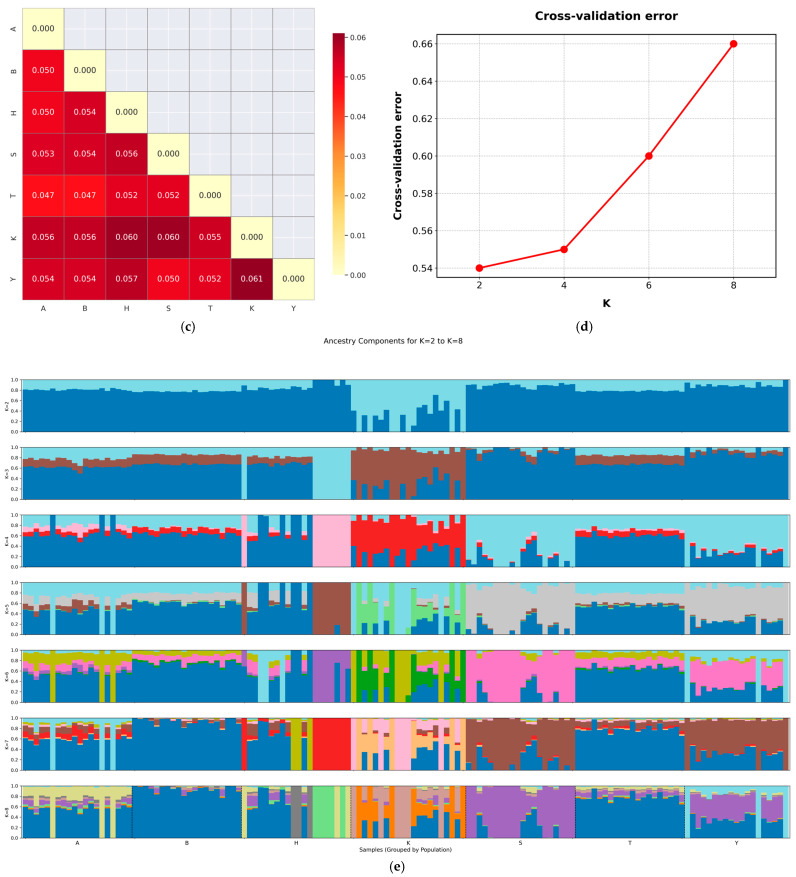
(**a**) Principal component analysis (PCA) of the seven populations; (**b**) Phylogenetic tree; (**c**) Genetic distances among the seven populations; (**d**) Cross-validation (CV) error corresponding to different K values; (**e**) Ancestral component analysis of the seven sheep populations.

Principal component analysis (PCA) was performed based on the obtained SNP markers. As shown in Figure 4a, the PC1/PC2 scatter plot clearly distinguishes Kirgiz sheep from the other populations, with Kazakh, Altay, Bayinbuluke, and Turpan Black sheep clustering together, while Bashibai and Yemule White sheep formed a distinct group. The PC1/PC3 plot (Figure 4a) confirmed this pattern, consistently separating Kirgiz sheep and highlighting their genetic distinctiveness among the seven Xinjiang populations analyzed.

### 3.4. Phylogenetic Tree Construction

To assess the degree of differentiation among sheep populations, genetic distances between individuals were calculated and a phylogenetic tree was constructed (Figure 4b). The phylogenetic tree of the seven local sheep populations showed that samples from each population clustered together without apparent admixture. Yemule White sheep and Bashibai sheep were genetically closely related, and Kazakh sheep and Altay sheep clustered on the same branch, also indicating close genetic relationships. These results were consistent with those obtained from the PCA.

### 3.5. Inter-Population Genetic Differentiation Index (F_ST)

The inter-population genetic differentiation index (F_ST) ranged from 0.047 to 0.061 (Figure 4c), showing an overall increasing trend with geographical distance. F_ST values were generally lower among the northern Xinjiang populations, which may reflect historically frequent gene flow, as well as relatively recent common ancestry and similar ecological environments. In contrast, the average F_ST between Kirgiz sheep from southern Xinjiang and other populations was as high as 0.061, significantly higher than the differentiation levels observed among northern Xinjiang populations. This pattern is likely related to their long-term habitation on the eastern edge of the Pamir Plateau, where gene flow has been restricted by mountainous terrain.

### 3.6. Population Structure Analysis

To further elucidate the genetic structure among sheep populations in Xinjiang, ADMIXTURE analysis was performed with the assumed number of ancestral populations (K) ranging from 2 to 8 (Figure 4e), and the corresponding cross-validation (CV) errors evaluated. The CV error reached its minimum at K = 2 (Figure 4d), indicating that this value best captured the underlying population structure. At K = 2, Kirgiz sheep exhibited a nearly homogeneous ancestry profile dominated by a single ancestral component, whereas populations from northern Xinjiang showed clear evidence of genetic admixture.

### 3.7. Selective Signature Analysis in Xinjiang Local Sheep Populations

#### 3.7.1. Independent Genetic Evolution of Kirgiz Sheep

**Figure 5:**
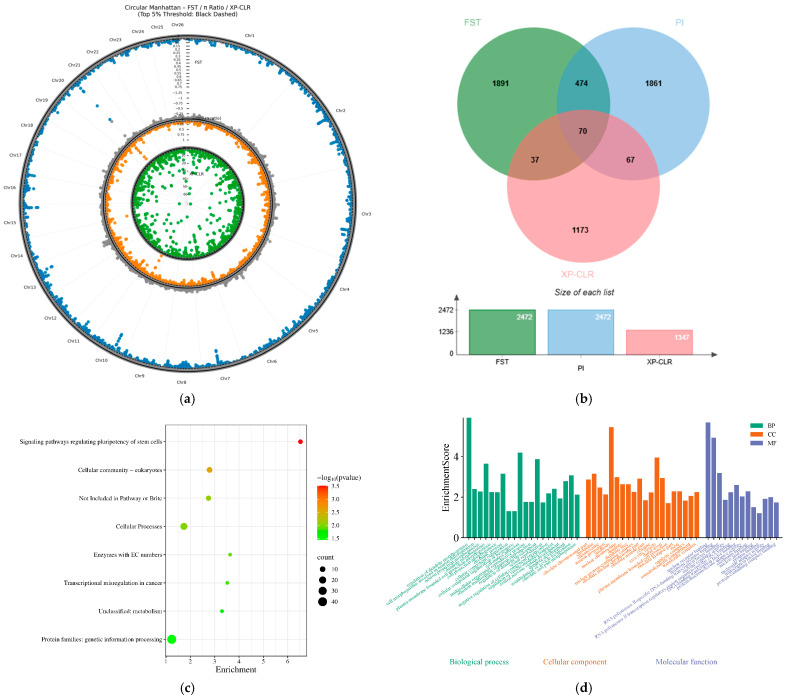
Selective signature analysis between Kirgiz sheep and the other six breeds; (**a**) Selective sweep Manhattan plots of F_ST, π ratio, and XP-CLR methods; (**b**) Overlapping Venn diagram of candidate regions identified by the three analyses; (**c**) KEGG enrichment analysis of candidate genes; (**d**) GO enrichment analysis of candidate genes.

Population genetic structure analysis indicated that Kirgiz sheep exhibited pronounced genetic differentiation from the other six local sheep populations in northern Xinjiang, forming an independent branch in the phylogenetic tree. This pattern is likely associated with their geographic distribution in the plateau mountainous regions of southern Xinjiang and long-term restricted gene flow. To elucidate their unique evolutionary trajectory, three selection signature detection methods—F_ST, π ratio, and XP-CLR—were comprehensively employed in a genome-wide scan by comparing genomic variations between Kirgiz sheep and the pooled six northern Xinjiang populations. As shown in Figure 5a, Manhattan plots generated by all three methods revealed strong selection signals across multiple chromosomal regions. By identifying overlapping candidate windows among the three methods (Figure 5b), a total of 70 high-confidence selected regions containing 102 candidate genes were identified. These signals were not detected in other populations, highlighting the uniqueness of the genomic evolutionary trajectory of Kirgiz sheep.

KEGG pathway analysis (Figure 5c) showed significant enrichment in “Signaling pathways regulating pluripotency of stem cells”, involving genes such as FSTL1, BMPR1B, PCGF1, and BMI1, suggesting that this pathway may play important roles in cell fate determination, tissue regeneration, and stress response, thereby contributing to the maintenance of physiological homeostasis in Kirgiz sheep under harsh high-altitude conditions. Additionally, the pathway “Cellular community—eukaryotes” was also significantly enriched and included genes such as BMPR1B, VTN, and PCGF1, indicating that reinforced intercellular connections may enhance tissue integrity to cope with environmental stresses, including drought, wind-sand erosion, and mechanical damage. GO functional analysis (Figure 5d) revealed that, at the molecular function level, “nuclear receptor binding” and “hormone receptor binding” were significantly enriched, with associated genes including NR1H3, NCOR1, and PCNA, indicating strong selection on endocrine signaling pathways involved in energy metabolism, reproductive regulation, and stress adaptation. At the cellular component level, “neuron projection” and “dendrite” were significantly enriched and involved genes such as NR1H3, SARM1, and STAU2, suggesting that specialization of nervous system structures may enhance perception and behavioral coordination in complex mountainous terrain. Meanwhile, “chromatin” and “nuclear chromosome” were significantly enriched, with genes including NCOR1, PCNA, NCOA1, and USP3, reflecting adaptive demands for maintaining genomic stability under intense ultraviolet radiation. At the biological process level, “regulation of dendrite morphogenesis,” “dendrite morphogenesis,” “dendrite development,” and “regulation of dendrite development” were all highly significantly enriched, underscoring the critical role of neuronal plasticity in adaptation to high-altitude environment. In addition, “cell—matrix adhesion” was significantly enriched, suggesting that strengthened extracellular matrix connections may help resist environmental pressures such as drought, wind-sand erosion, and mechanical damage. In summary, under the dual selection pressures of long-term geographic isolation and extreme plateau environments, the genome of Kirgiz sheep has undergone directional selection in key functional modules—including cell adhesion, neural development, genomic stability, and hormone signaling—resulting in a unique genetic adaptation system distinct from those of other local sheep populations in Xinjiang. This adaptive system provides a solid molecular foundation for their survival and reproduction in the southern Xinjiang plateau.

#### 3.7.2. The Adaptive Genetic Basis of Heat Tolerance

**Figure 6:**
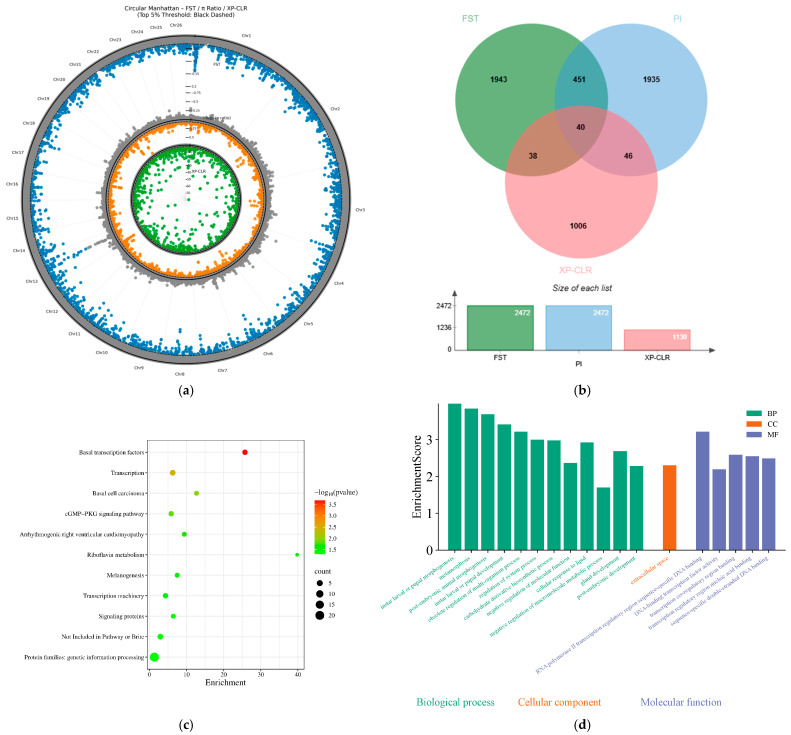
Selective signature analysis between heat-tolerant and non-heat-tolerant groups; (**a**) Selective sweep Manhattan plots of F_ST, π ratio, and XP-CLR methods; (**b**) Overlapping Venn diagram of candidate regions identified by the three analyses; (**c**) KEGG enrichment analysis of candidate genes; (**d**) GO enrichment analysis of candidate genes.

To elucidate the genetic mechanisms underlying heat tolerance in sheep, this study used Turpan Black sheep—long adapted to the extreme arid and hot environment of the Turpan Basin—as the heat-tolerant experimental group in this study. A pooled group of Altay and Bayinbuluke sheep, which share a relatively close genetic background but have long been adapted to cold alpine environments, was selected as the cold-adapted control group. This contrast between extreme thermal environments facilitates the identification and enrichment of genetic variants associated with temperature adaptation. By integrating three selection signature detection methods, significant selection signals were detected across multiple chromosomal regions, as shown in the Manhattan plots generated by all three approaches (Figure 6a). Intersecting the candidate regions identified by the three methods (Figure 6b) yielded 40 overlapping candidate windows, corresponding to 75 candidate genes. Of these, 45 genes with clear functional annotations were retained as high-confidence candidates for downstream analyses.

KEGG pathway enrichment analysis (Figure 6c) revealed that the candidate genes were significantly enriched in multiple pathways. Notably, the “Basal transcription factors” and the “cGMP-PKG signaling pathway” pathways showed significant enrichment, suggesting that under high-temperature stress, cells may maintain gene expression fidelity and protein homeostasis through modulation of core transcriptional machinery and second messenger signaling pathways. Accordingly, GTF2I, GTF2IRD1, and PRKCE were identified as potential key genes contributing to heat tolerance. In addition, enrichment of the “Melanogenesis” pathway—mediated by genes such as MC1R and TCF7L2—likely reflects morphological and epidermal adaptations to intense solar radiation in the Turpan Basin. GO enrichment analysis (Figure 6d) further revealed significant overrepresentation of molecular functions related to transcriptional regulation. Specifically, “DNA-binding transcription factor activity” and “RNA polymerase II transcription regulatory region sequence-specific DNA binding” were both significantly enriched, involving genes including TCF7L2, GTF2I, GTF2IRD1, TBPL1, TCF21, EBF4, and the histone variant H2AZ1. These suggest a coordinated role for these factors in transcriptional reprogramming under heat stress. Moreover, the biological process term “cellular response to lipid” was significantly enriched, with associated genes (PTCH1, TUT4, TCF21, PRKCE, H2AZ1) implicating lipid-mediated stress responses in thermotolerance. Notably, several negative regulatory processes—including “negative regulation of macromolecule metabolic process”—were also significantly enriched. This indicates that under heat stress conditions, cells may enhance environmental adaptability by selectively suppressing non-essential biosynthetic activities to reallocate energy resources toward critical survival functions.

#### 3.7.3. The Genetic Basis of Body Size Differentiation

**Figure 7:**
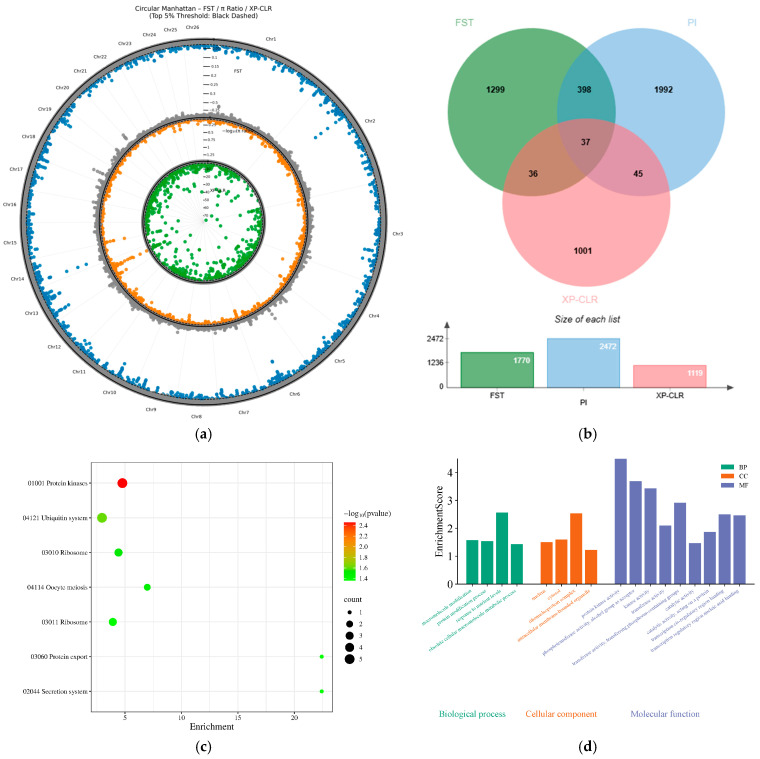
Selective signature analysis related to body size differentiation; (**a**) Selective sweep Manhattan plots of F_ST, π ratio, and XP-CLR methods; (**b**) Overlapping Venn diagram of candidate regions identified by the three analyses; (**c**) KEGG enrichment analysis of candidate genes; (**d**) GO enrichment analysis of candidate genes.

To investigate the genetic basis of body size differentiation in sheep, a pooled group of larger-bodied breeds (Altay sheep and Bashibai sheep) was designated as the experimental group, whereas a pooled group of smaller-bodied breeds (Turpan Black sheep and Bayinbuluke sheep) was used as the control group. This comparative design aimed to identify candidate genes under positive selection in large-bodied sheep populations that may be involved in growth and developmental processes. By integrating three selection signature detection methods (Figure 7a), a total of 37 genomic regions under selection were identified (Figure 7b), encompassing 61 genes. Among these, 45 functionally annotated candidate genes were retained for downstream analyses.

KEGG pathway analysis (Figure 7c) revealed significant enrichment in two major pathways: “Protein kinases,” which included RIOK3, BUB1B, TRIM24, KIT, and EIF2AK4, and the “Ubiquitin system”, which was enriched with RNF11, TRIM24, HERC6, and ZBTB46. GO functional enrichment analysis further confirmed that these candidate genes were significantly overrepresented in multiple biological processes and molecular functions. As shown in Figure 7d, at the biological process level, “macromolecule modification” (GO:0043412) and “response to nutrient levels” (GO:0031667) were significantly enriched. At the molecular function level, “protein kinase activity” (GO:0004672), “kinase activity” (GO:0016301), and “transcription cis-regulatory region binding” (GO:0000976) exhibited high significance. notably, genes involved in transcriptional regulation—including CUX1, BNC2, ZGPAT, SLC2A4RG, and TRIM24—were associated with the relevant GO terms. Genes exhibiting protein kinase activity included RIOK3, BUB1B, KIT, TRIM24, and CUX1. In addition, RNF11, HERC6, and TRIM24 were implicated in ubiquitin-mediated protein modification processes. In summary, candidate genes associated with body size differentiation in sheep are predominantly enriched in core biological processes, including transcriptional regulation, protein kinase-mediated signal transduction, and ubiquitin–proteasome-dependent protein degradation. These findings suggest that coordinated regulation of these pathways may constitute the molecular basis underlying phenotypic variation in body size among different sheep breeds.

## 4. Discussion

### 4.1. Analysis of Population Genetic Structure in Xinjiang Local Sheep

Livestock germplasm resources constitute a vital strategic asset for safeguarding agricultural security, and their genetic diversity provides the fundamental basis for the sustainable development of animal husbandry [18]. In this study, analyses of genetic diversity across seven Xinjiang local sheep populations revealed that observed heterozygosity (Ho) was consistently greater than or equal to expected heterozygosity (He), with the proportion of polymorphic markers ranging from 0.9699 to 0.9802. This pattern of heterozygote excess may reflect technical artifacts related to sample size or effective avoidance of consanguineous mating in managed flocks. Long-term monitoring integrating temporal samples and pedigree data would be required to rigorously assess conservation effectiveness and genetic integrity over time.

The complex topography and pronounced climatic gradients of Xinjiang have imposed unique natural selection pressures, shaping the phylogenetic relationship and adaptive evolution of local sheep breeds [19]. Principal component analysis (PCA) revealed a clear genetic separation between the Kirgiz sheep from southern Xinjiang and all northern Xinjiang populations, whereas the northern groups exhibited close genetic affinities. This pattern was further confirmed by pairwise F_ST analyses, which showed that genetic differentiation increased with geographic distance, with the highest F_ST values observed between Kirgiz sheep and the northern Xinjiang populations, indicating the greatest level of genetic divergence. Kirgiz sheep are primarily distributed in the high-altitude mountainous regions of southern Xinjiang, where surrounding mountain ranges from natural geographic barriers that severely limit gene flow with other populations. Over time, this prolonged isolation has driven the emergence of distinct evolutionary trajectories and unique genetic characteristics in this breed [20]. The underlying molecular mechanisms warrant further investigation through functional genomics approaches. In contrast, sheep populations from northern Xinjiang exhibit minimal genetic differentiation, likely due to their relatively recent shared ancestry and historically frequent gene flow. For instance, the seasonal transhumance practices of Kazakh herders have facilitated extensive genetic exchange among sheep flocks across different pastures in northern Xinjiang. Conversely, the traditionally sedentary grazing system of the Kirgiz ethnic group in southern Xinjiang may have reinforced the genetic consequences of geographic isolation at the socio-cultural level.

### 4.2. Analysis of Unique Adaptive Molecular Mechanisms in Kirgiz Sheep Under the Background of Genetic Differentiation

Population genetic analyses revealed a pronounced genetic differentiation between Kirgiz sheep and other Xinjiang sheep populations. Based on genome-wide selective sweep scanning, a systematic and multi-layered adaptive genetic regulatory network was identified in Kirgiz sheep. These strong selection signals are consistent with the extreme environmental pressures of the Pamir Plateau— characterized by severe cold, hypoxia, and intense ultraviolet radiation—suggesting that these factors may have driven directional genomic selection in this population, ultimately leading to their genetic divergence from other regional sheep breeds.

In the context of intense high-altitude ultraviolet radiation, several genes associated with DNA damage recognition and repair exhibited strong signatures of positive selection, suggesting that alleles at these loci may have increased in frequency due to selective pressures related to UV exposure. The DDB2 gene, which has been implicated in recognizing UV-induced DNA lesions and enhancing the activity of DNA repair complexes, may contribute to efficient DNA repair processes [21,22]. Concurrently, PCNA, a core platform protein involved in DNA replication and repair, was also under selection. By forming complexes with proteins such as POLD1, PCNA could directly regulate DNA synthesis and DNA damage repair pathways [23]. Faced with the combined challenges of hypoxia and low temperatures, Kirgiz sheep also displayed adaptive optimization in energy metabolism and oxygen utilization. SLC25A4, which functions in mitochondrial energy conversion, was among the selected genes, suggesting a possible role in maintaining energy homeostasis under hypoxic conditions [24]. The oxygen release regulation mediated by the ADORA2B receptor [25], together with the roles of FSTL1 in pulmonary vascular remodeling and cardiac tissue protection [26,27], as well as the cardioprotective function of NCoR1 during ischemic injury [28], may collectively constitute an adaptive foundation for cardiovascular and respiratory system function. Furthermore, to maintain tissue homeostasis and regenerative capacity under harsh environmental conditions, the pathway “Signaling pathways regulating pluripotency of stem cells” showed significant enrichment. This pathway includes key genes like BMPR1B, PCGF1, and BMI1. BMPR1B, as a bone morphogenetic protein receptor, could activate the p38 MAPK signaling pathway to promote cell proliferation, thereby potentially playing an important role in tissue repair and homeostasis maintenance [29]. PCGF1 and BMI1 function as epigenetic regulators and may contribute to maintaining stem cell self-renewal and pluripotency [30,31]. Enrichment of cell adhesion-related genes, such as VTN, also suggests reinforced cell–cell interactions and tissue structural integrity. VTN participates in cell adhesion and tissue damage repair processes, with studies showing its close correlation with complement system regulation under low-temperature stress in fish [32]. In terms of antioxidant defense, PRDX6 has been reported to contribute to the clearance of excessive reactive oxygen species (ROS) and facilitate repair of oxidatively damaged cell membranes, potentially protecting cardiovascular and pulmonary vascular endothelial function and maintaining tissue homeostasis [33]. The mitochondrial serine protease HTRA2 helps maintain mitochondrial protein homeostasis and clear misfolded proteins, further ensuring cellular energy and stress response capabilities [34]. Notably, adaptation mechanisms also extend to energy storage and neural regulation. The fat deposition-related CDS2 gene may enhance the body’s energy storage and insulation capabilities against cold [35]. Enrichment of nervous system development-related pathways and selection of genes like NLGN1 and FRMD4A suggest potential adaptive evolution in perception and behavioral neural functions [36,37]. In summary, adaptation of Kirgiz sheep to the extreme environmental condition of the Pamir Plateau does not depend on the effect of a single gene, but instead involves a highly integrated and coordinated network of multiple biological processes, including DNA damage repair, hypoxia-adaptation energy metabolism, and cardiovascular regulation. The selection signatures retained in the genome systematically elucidate the molecular basis of the unique adaptive traits in this population and provide a solid genetic foundation for their long-term survival and reproduction in the harsh plateau environment. However, the precise functional interactions within this network remain to be experimentally validated.

### 4.3. Genetic Adaptation Mechanisms Under Extreme Temperature Selection Pressure

During their long-term evolutionary process, livestock are subjected to intense natural selection imposed by climatic environments, with temperature acting as a key ecological factor. The Turpan Basin, as a typical hyper-arid and hot region, provides an ideal natural model for dissecting heat tolerance in sheep owing to its unique climatic conditions. Through genome-wide positive selection signature scanning, this study identified a series of significantly differentiated candidate genes between Turpan Black sheep and Altay sheep as well as Bayinbuluke sheep, revealing multi-layered molecular strategies for coping with extreme heat stress.

First, at the level of basal transcriptional regulation, heat tolerance-related genes were significantly enriched in core pathways such as “Basal transcription factors” and “Transcription”, involving key factors including GTF2I, GTF2IRD1, and TBPL1. GTF2IRD1 is a member of the GTF2I gene family, which encodes a group of multifunctional transcription factors [38]. The GTF2I gene has been implicated in neural development and cardiovascular homeostasis [39], suggesting that Turpan Black sheep may optimize the intrinsic efficiency of the transcriptional apparatus to counteract potential transcriptional stalling or RNA polymerase inactivation induced by high temperatures, thereby ensuring continuous and stable expression of essential genes under heat stress. At the level of cellular stress defense, the genes PRKCE and DNAJB14 exhibited strong selection signals. PRKCE may function as a key regulator linking lipid metabolism with cellular stress response. Regulation of PRKCE can influence fatty acid metabolic flux, thereby reducing harmful byproducts such as reactive oxygen species generated during mitochondrial β-oxidation and protecting cells from oxidative damage under metabolic stress [40,41]. This gene has been confirmed to participate in heat stress responses in Chinese Holstein cows [42], further supporting its conserved role in ruminant heat tolerance. Simultaneously, DNAJB14, a member of the type II transmembrane Hsp40 protein family, has been shown to collaborate with HSP70 to assist in the proper folding of nascent polypeptide chains as well as the repair or degradation of misfolded proteins, potentially counteracting heat-induced protein denaturation and aggregation and may contribute to comprehensive defense system against proteotoxic stress such as heat exposure [43,44]. Its significant upregulation in patients with hemorrhagic stroke and heat-stasis syndrome further supports its protective role in heat stress response from a human disease perspective [45]. At the level of physical barrier function and morphological adaptation, multiple candidate genes indicated optimization of barrier integrity and physical protection in Turpan Black sheep. SPINK7, a serine protease inhibitor expressed in epithelial tissues, has been associated with inflammatory skin diseases [46] and may enhance resistance to arid-hot environments by regulating skin inflammatory responses and barrier integrity. The genes MC1R and TCF25, which are known as key regulatory factors determining coat color, have been reported to modulate hair and skin pigmentation as well as melanin production [47,48]. These genes are likely associated with the dark coat phenotype observed in Turpan Black sheep. The abundant melanin present in dark coats may effectively absorb and block intense ultraviolet (UV) radiation, thereby mitigating UV-induced damage to the skin and underlying tissues. This mechanism likely constitutes a direct physical protective strategy for coping with high-radiation environments and alleviating heat stress [49]. Additionally, selection signals detected at FAM117B suggest the cardiovascular system may also have undergone adaptive selection. This gene was found to be significantly upregulated in a pathological cardiac hypertrophy mouse model, and its expression level was positively correlated with key molecules in the cAMP signaling pathway [50]. Under persistent high-temperature conditions, increased heart rate and circulatory load in animals can induce compensatory myocardial hypertrophy; therefore, selection acting on this gene may contribute to enhanced cardiac tolerance to long-term heat stress. In summary, the evolution of heat tolerance in Turpan Black sheep represents a complex, multi-layered, and synergistic process. This adaptation does not depend on the effect of a single gene but instead involves the establishment of an efficient internal homeostasis defense system through optimization of basal transcriptional efficiency to ensure gene expression stability, enhancement of protein folding capacity and oxidative damage defenses to maintain cellular function, and improvement of physical barriers and systemic physiological functions. Collectively, this comprehensive genetic adaptation strategy enables Turpan Black sheep to maintain physiological robustness and survival capacity in extreme arid-hot environments.

### 4.4. Mechanisms of Growth and Development-Related Genes

Sheep body weight and body size, as composite phenotypes, result from the coordinated effects of multiple traits including skeletal framework, muscle mass, and fat deposition. To elucidate the genetic basis of body size variation, this study compared selective signatures between larger-bodied sheep (Altay and Bashibai) and smaller-bodied sheep (Turpan Black and Bayinbuluke), thereby identifying a set of candidate genes associated with growth and development. Functional enrichment analysis revealed that these genes do not act in isolation but are systematically enriched in key pathways, including transcriptional regulation, protein kinase activity, and ubiquitin-mediated modification. Together, these pathways form a multi-layered regulatory network spanning from gene expression programming to protein function execution, providing a systematic framework for understanding the molecular mechanisms of body size formation.

First, fine-tuning at the transcriptional level plays a central role in body size formation. Key transcription factors, including BNC2, CUX1, MAML3, TRIM24, and KIT, have been suggested to form a core regulatory network. BNC2, a DNA-binding transcriptional regulator, plays an important role in mesenchymal cell development, and previous studies have demonstrated its involvement in muscle tissue differentiation and development in Sunit Bactrian camels [51]. CUX1 appears to play a key role in regulating cell proliferation, differentiation, and the establishment of tissue and organ size. Its functional roles have been demonstrated in the reproductive development of rhesus monkeys and human cerebral cortex development [52,53], and recent studies further reveal its ability to promote brown adipocyte differentiation and lipid droplet accumulation [54]. Notably, MAML3, a transcriptional coactivator of the Notch signaling pathway, directly influences organ morphogenesis by regulating the proliferation of cardiomyocytes and chondrocytes [55,56]. Together, these factors form a transcriptional hub responsive to internal and external environmental signals, thereby systematically defining growth potential. Second, at the level of signal transduction and protein modification, this regulatory module includes genes such as RIOK3, BUB1B, EIF2AK4, and KIT. These kinases act as molecular switches that rapidly transmit extracellular signals, such as growth hormone and insulin, through phosphorylation events, thereby precisely regulating the cell cycle, metabolism, and growth rate. RIOK3, an RNA-binding protein kinase, may contribute to skeletal development by coordinating protein synthesis (ribosomal function) and immune signaling (interferon pathway), offering a novel perspective for understanding the genetic basis of skeletal architecture related to body size [57]. BUB1B has been implicated in regulating tissue growth through its involvement in cell cycle regulation [58]. Regarding protein stability regulation, RNF11 is significantly upregulated during the osteogenic differentiation of bone marrow mesenchymal stem cells, where it has been suggested to play a critical role in skeletal system development and repair [59]. Meanwhile, HERC6 was found to be significantly associated with adult live weight in Uruguayan Merino sheep [60], indicating that E3 ubiquitin ligases may provide precise regulation for body size development by modulating the degradation of key regulatory proteins. KIT is a well-known pleiotropic gene that plays essential roles not only in coat color formation through melanocyte regulation but also in muscle development. In the context of body size differentiation, KIT is the stem cell factor receptor. Studies show that KIT, as a target gene of MITF, can promote myofiber hypertrophy and protein accumulation via the MITF–MAPK axis [61]; its expression level is positively correlated with myofiber size and muscle development in pigs and ducks [62,63]. Combined with its positive selection in larger-bodied sheep, these findings suggest that KIT may contribute to increased body size by promoting myofiber growth. The CUL7 gene is a core component of the E3 ubiquitin ligase complex. Studies have shown that cartilage-specific knockout of CUL7 in mice leads to significant growth restriction and markedly increased chondrocyte apoptosis; in vitro experiments also have further confirmed that Cul7 deletion promotes chondrocyte death, thereby interfering with endochondral ossification [64]. These findings suggest that CUL7 may play a key regulatory role in longitudinal bone growth by maintaining chondrocyte survival and normal ossification. Furthermore, some candidate genes, although not reaching statistical significance in the enrichment analysis, may still contribute to body size regulation based on their known biological functions. For example, SVOPL, a member of the solute carrier family, has been associated with backfat thickness and carcass weight in pigs [65], suggesting that it may indirectly influence tissue growth by regulating cellular nutrient uptake or metabolite efflux. MYO3B, a myosin family member involved in cytoskeletal organization and cell motility, has been associated with growth traits in sheep and carcass weight in pigs [66,67]. STMN3, a microtubule-depolymerizing protein that regulates mitosis and morphogenesis, has been reported to affect body height in pigs [68]. MEGF11 shows a significant correlation with body weight in Inner Mongolia Cashmere goats and may be involved in energy utilization and nutrient allocation [69]. TNFRSF6B has been shown to promote myoblast proliferation and plays a direct role in muscle development [70]. The lipid-binding protein gene OSBPL9 may function in lipid metabolism and growth or development [71], whereas ATRN, a key receptor in the ASIP signaling pathway, may influence fat deposition and body size formation by regulating energy homeostasis and lipid metabolism [72]. In summary, the genetic differentiation underlying sheep body weight and body size originates from a multi-level, coordinated regulatory system encompassing skeletal growth patterning, muscle development promotion, and fat metabolism regulation. Collectively, these mechanisms translate genetic variation into growth phenotypes, not only elucidating the molecular basis of size divergence between large and small sheep breeds but also providing a systematic perspective on the complex genetic architecture underlying mammalian body size evolution.

## 5. Conclusions

Based on whole-genome data from seven local sheep populations in Xinjiang, this study systematically analyzed their genetic diversity, population structure, and adaptive evolutionary mechanisms. The results indicated detectable levels of within-population genetic diversity in all populations, and that geographic isolation is the primary factor driving genetic differentiation. Notably, significant genetic isolation was observed between Kirgiz sheep from the Pamir Plateau in southern Xinjiang and sheep populations from northern Xinjiang. Selection signal analysis revealed that Kirgiz sheep have undergone coordinated selection in pathways related to stem cell pluripotency regulation, DNA repair, and neural development. These adaptive traits likely constitute an important molecular basis for the ability of Kirgiz sheep to adapt to the extreme plateau environment and, consequently, to maintain distinct genetic differentiation from other populations. Heat adaptation of Turpan Black sheep was associated with mechanisms including transcriptional regulation, protein homeostasis maintenance, and melanin synthesis. Comparative analysis of body size identified a set of development-related candidate genes, whose functions are enriched in pathways including transcriptional regulation, kinase signaling, and the ubiquitin system. These genes collectively participate in regulating the development of skeletal, muscular, and adipose tissues, thereby forming a multi-layered genetic network underlying body size differentiation. It should be noted that these findings are based on indirect population genetic inferences, our sample size is limited, and the functional roles of the candidate genes require further biological validation. From a genomic perspective, this study systematically elucidates the population genetic structure, ecological adaptation mechanisms, and genetic basis of important economic traits in the seven local sheep populations from Xinjiang, providing practical guidance for precision breeding and genetic resource conservation strategies for Xinjiang indigenous sheep.

## Figures and Tables

**Table 1:** Sample collection from the seven populations.

Population	Abbreviation	Sampling Location	Sample Size
Altay sheep	A	Fuhai County, Altay Prefecture	20
Bayinbuluke sheep	B	Hejing County, Bayingolin Mongol Autonomous Prefecture	20
Kazakh sheep	H	Tekes County, Ili Kazakh Autonomous Prefecture	20
Kirgiz sheep	K	Akto County, Kizilsu Kirgiz Autonomous Prefecture	21
Bashibai sheep	S	Yumin County, Tacheng Prefecture	20
Turpan Black sheep	T	Toksun County, Turpan City	20
Yemule White sheep	Y	Emin County, Tacheng Prefecture	19

## Data Availability

The data presented in this study are available within the article.
